# DNA vaccines: designing strategies against parasitic infections

**DOI:** 10.1186/1479-0556-2-17

**Published:** 2004-12-03

**Authors:** Catherine Ivory, Kris Chadee

**Affiliations:** 1 Institute of Parasitology of McGill University, Macdonald Campus, 21,111 Lakeshore Road, Ste. Anne de Bellevue, Quebec, Canada, H9X 3V9

## Abstract

The complexity of parasitic infections requires novel approaches to vaccine design. The versatility of DNA vaccination provides new perspectives. This review discusses the use of prime-boost immunizations, genetic adjuvants, multivalent vaccines and codon optimization for optimal DNA vaccine design against parasites.

## Introduction

DNA vaccination was introduced in 1990 by a study that demonstrated the induction of protein expression upon direct intramuscular injection of plasmid DNA in myocytes [1]. DNA vaccines are new types of sub-unit vaccines allowing protein expression in mammalian cells after introduction of plasmid or recombinant viral vectors encoding the selected protective antigen. Protective immunity conferred by DNA vaccines has been shown in many animal models of various diseases including HIV, tuberculosis and cancer [2-4]. DNA vaccines induce strong humoral and cellular immunity and have the potential to increase immunogenicity through modifications of the vector or incorporation of adjuvant-like cytokine genes.

Successful vaccines should be able to induce strong immune responses which are long-lasting and in most cases providing protection against different strains of the same pathogen. Progress has been made towards development of DNA vaccines against viral and bacterial pathogens showing protection and lasting immunity [5]. Application of this new vaccination technology with regard to parasitic infection provides new hope for significant advances in anti-parasitic vaccine research. An important consideration in developing vaccines against parasites is the complexity of parasitic diseases. Parasite infections, unlike most viral and bacterial infections, tend to be chronic and associated with immunodepression or inappropriate immune responses [6]. Parasites have complex life cycles and host immunity to stage-specific antigens may not overlap with other later stages or vector-borne stages. Antigenic variation and other immune evasion mechanisms also complicate the development of vaccines against parasites. However, with recombinant DNA technology and the versatility of DNA vaccination, it is now possible to take rational parasite specific strategies to vaccine design and overcome the obstacles presented by parasitic diseases. Improving DNA vaccine efficacy against parasitic disease can be achieved by: prime-boost immunizations, genetic adjuvants, multivalent vaccines or codon optimization. This review describes the application of these strategies, using specific parasites as examples, to improve DNA vaccine efficacy (see Table 1[7-19]).

**Table 1:** Summary of DNA vaccine optimization in parasites

**Optimization Method**	**Parasite**	**Specific Modifications and Improved Responses**	**Reference**
**Genetic Adjuvant**	Malaria	Co-immunization of merozoite surface protein-1 (MSP1) of *P. yoelii *with IL-12 in A/J mice elicited strong Th1 type responses characterized by high levels of IFN-γ. Parasite specific antibodies also protected against parasite infection.	[7]
		Construction of DNA plasmid encoding C-terminal region of MSP1 (*P. falciparum*) was tested with plamids expressing GM-CSF or recombinant GM-CSF protein in monkeys. Co-immunization with GM-CSF protein lead to higher Ab titers and higher response to boosting with MSP1.	[8]
		MuStDO5 is a multivalent vaccine composed of 5 plasmids encoding *P. falciparum *proteins and GM-CSF. When tested for safety in mice and rabbits via i.m/i.d. injections, the vaccine was determined safe and well tolerated without development of autoimmunity.	[9]
	Leishmania	Vaccination with plasmids encoding *L. amazonensis *P4 nuclease, HSp70 or murine IL-12 was tested in the susceptible Balb/c mouse model. Co-immunization with P4 nuclease and IL-12 protected mice against parasite challenge as determined by 4 log reduction in parasite burden and increased levels of IFN-γ and TNF-α.	[10]
		Following p36/LACK prime-boost immunization with a combination of DNA vectors expressing IL-12 and IL-18 in mice, highest protection was observed compared to controls.	[11]
	Schistosoma	Co-administration of DNA plasmids encoding IL-18 and *S. mansoni *glutathione S-transferase elicited 30 fold increase in antigen specific IFN-γ secreting cells, 28% reduction in egg laying and 23% reduction in worm burden in mice.	[12]
**Multivalent vaccine**	Malaria	Prime boost regimen with vectors encoding functional domains of TRAP and CS antigens of *P. cynomogli *was more effective at reducing peak parasitemia in rhesus monkeys.	[13]
		A multistage *P. knowlesi *vaccine with plasmids encoding 2 pre-erythrocytic, 2 blood stage antigens and GM-CSF was administered to rhesus monkeys followed by a boost with a pox virus encoding all 4 antigens. Monkeys developped Abs against sporozoites, infected erythrocytes and CPS protein.	[14]
		Six pre-erythrocytic antigens linked together to produce a polyprotein in a DNA vaccine and either MVA or FP9 were tested in mice against *P. falciparum*. Greater responses were seen when a heterologous viral regimen was used, producing multispecific T cells.	[15]
	Leishmania	*L. major *TSA and LmST11 antigens were expressed either as single genes or as digene construct and tested in the susceptible Balb/c model. Administration of the genes in either constructs lead to protection via polyspecific immune responses.	[16]
	Schistosoma	Three doses of 4 plasmids encoding *S. japonicum *antigens, Sj62, Sj28, Sj23 and Sj14 3-3-, induced high levels of IFN-γ and partial protection from challenge infection when administered in mice.	[17]
	Entamoeba	DNA plasmids encoding either *Entamoeba histolytica *cysteine protease 112 or adhesin 112 were co-administered to hamsters, leading to protection against liver abscess formation. No protection was observed with either plasmid alone.	[18]
**Codon optimization**	Malaria	*P. falciparum *erythrocyte binding protein and MSP1 antigens were codon optimized for expression in mammals. 10 to 100 fold less optimized plasmid DNA was required to induce high Ab titers in mice.	[19]

## Prime-Boost Immunizations

Current sub-unit vaccines predominantly induce strong antibody responses and weak cellular immunity. DNA vaccines in animal models can induce both strong humoral and cellular mediated responses, but although safe in humans, DNA vaccines do not produce the same magnitude of cellular immunity [20]. In cases where the pathogen is intracellular, an antibody response is not sufficient for protection and cell-mediated immunity is required. This is the case with malaria, where the parasite infects hepatocytes and erythrocytes, and cytotoxic T cells play an important role in protection. Therefore, it is important to devise vaccination strategies that enhance T cell immunogenicity and confer a protective cellular immune response to intracellular pathogens. A novel approach to increase T cell responses to vaccination is the heterologous prime-boost immunization strategy [21]. This method consists of priming and boosting with different vectors encoding the same antigen. The principle of the strategy is to first prime some T cells to be antigen-specific and then boost to induce rapid T cell expansion upon repeated exposure to the specific antigen. DNA plasmids are good priming agents since they are internalized by antigen presenting cells and can induce antigen presentation via MHC class I or class II. DNA plasmid backbones are immunogenic due to the presence of stimulatory unmethylated CpG motifs that readily induce Th1 cytokine expression, leading to cellular mediated immunity. Recombinant viral vectors, which are non-replicating and safe, are excellent for boosting. Viral vectors induce high protein expression and presentation via MHC class I which leads to greater antigen specific T cell expansion [22]. Common boosting vectors in vaccine trials include modified Vaccinia virus Ankara (MVA), recombinant Vaccinia virus (rVv), attenuated adenoviruses, and attenuated pox viruses like fowl pox (FP9). These viruses are highly attenuated and non-replicating but still able to produce proteins. The MVA vector, for example, was developed by over 500 serial passages in chicken embryo fibroblasts and has acquired a replication defect in late stage virion assembly. This vector was used for smallpox vaccinations in 1970 and is known to be safe as well as highly immunogenic. Viral vectors induce strong production of proinflammatory cytokines, which generate greater levels of cell-mediated immunity. Overall the immunogenicity of viruses is greater than that of plasmid DNA, however when administered alone the immune response is generally targeted to vector components. For this reason heterologous vaccination, priming and boosting with different vectors, promotes antigen-specific responses rather than vector-specific responses. The resulting effect when using the heterologous prime-boost technique is the generation of memory T cells to the antigen by priming then amplification of these cells by boosting. This approach has been used extensively to create effective immunizations against malaria, and in a variety of parasites [23-32] (see Table 2).

**Table 2:** Prime-boost immunization trials against parasites

**Parasite**	**Antigen**	**Priming agent**	**Boosting agent**	**Response**	**Reference**
**Malaria**	Circumsporozoite protein of *P. berghei*	Attenuated fowlpox virus or DNA	MVA	Potent CD8+ T cell responses were elicited in mice with FPV/MVA vaccination. Novel regimen was more protective against challenge than DNA-MVA immunizations.	[7]
	*P. falciparum *surface protein (Pfs25)	DNA	Recombinant protein	Intramuscular injections in rhesus monkeys showed significant increase in transmission blocking antibodies.	[8]
	Circumsporozoite protein of *P. yoelii*	DNA	Pox virus	Immunized neonatal mice showed 93% protection which was CD8+ T cell dependent.	[9]
	*P. falciparum *erythrocyte binding protein	DNA	Recombinant protein	Higher antibody titers and the ability to reduce parasitemia without drug intervention in Aotus monkeys.	[10]
	Circumsporozoite protein of *P. falciparum*	DNA	RTS, S/ASOZA	Malaria volunteers develop *P. falciparum *specific Abs and Th1 specific CD4+ and CD8+ T cells upon vaccination.	[11]
**Leishmania**	*Leishmania infantum *LACK	DNA	Recombinant vaccinia virus	60% protection, associated with cell mediated responses, was observed in dogs after challenge compared to controls.	[12]
	p36/LACK	DNA	Recombinant vaccinia virus	Vaccination in mice resulted in 70% reduction in lesion size and 1000-fold reduction in parasite loads.	[13]
	*L. infantum *acidic ribosomal protein PO (LiPO)	DNA	Recombinant protein	Boosting elicited stronger IgG2a titers but could not protect against challenge compared to DNA alone.	[14]
**Schistosome**	Cu/Zn cytosolic superoxide dismutase (SOD), signal peptide SOD and glutathione peroxidase (GP)	DNA	MVA	DNA vaccines were tested against *S. masoni *challenge in mice. Boosting with MVA for the same genes had no increased effect expect for mutated GP antigen were boosting lead to 85 % protection.	[15]

To further improve the efficacy of a *Plasmodium yoelii *DNA vaccine, mice were primed intramuscularly with DNA vaccine and granulocyte/macrophage colony stimulating factor (GM-CSF) plasmid and boosted with rVv encoding the same circumsporozoite protein (CSP) [33]. This combined strategy of genetic adjuvant and prime-boost immunization elicited improved responses and protection while also reducing the dose of initial DNA vaccine required. In chimpanzees, a DNA-prime and MVA-boost regimen encoding thrombosin-related adhesion protein (TRAP) with GM-CSF protein as adjuvant induced specific T cell and antibody response that was long lasting against *P. falciparum *[34]. Complete protection against *P. berghei *challenge characterized by strong CD8+ T cell responses was observed in mice after intradermal adenovirus-prime-MVA-boost encoding CSP [35]. These studies led to the assessment of prime-boost immunizations in humans in both naive volunteers and field trials in endemic areas. DNA-prime-MVA-boost vaccines encoding a polyepitope string fused to *P. falciparum *pre-erythrocytic TRAP antigen were administered via gene-gun to healthy volunteers with no adverse effects [36]. The polyepitope in the vaccine encodes a single polypeptide, which constitutes of a string of T and B cell epitopes from different sources, including tetanus toxin and BCG. In fact, this heterologous prime-boost immunization elicited interferon-γ (IFN-γ) secreting, antigen-specific T cells in humans, which were significantly higher than responses observed with either vector alone [37]. Furthermore, this study demonstrated partial protection, measured by delayed parasitemia, after challenge with a different strain of *P. falciparum*. Another group demonstrated that priming with DNA vaccine for *P. falciparum *CSP and boosting with a recombinant protein vaccine in adjuvant (RTS, S/AS02A) induced the production of significant antibody and T cell responses in healthy volunteers [38]. Phase I clinical trials in The Gambia in semi-immune adults have demonstrated that heterologous DNA-prime-MVA-boost regimen encoding *P. falciparum *TRAP antigen is safe, well tolerated and induces responses greater than those observed in naive volunteers [39]. Boosting with the MVA vaccine 12 months after the initial prime-boost immunization in this clinical trial was successful in re-expanding the T cell population and demonstrated the safe use of MVA to boost at different periods to maintain T cell immunity.

## Genetic Adjuvants

Adjuvants are used to strengthen the immune response to a vaccine and have been critical in modern vaccine development. Genetic adjuvants are expression vectors encoding biologically active molecules such as cytokines, chemokines and co-stimulatory molecules. These adjuvants can be encoded on the same vector as the antigen or expressed on a separate vector and co-injected with the vaccine. This method provides adjuvant activity at the site of antigen production, with lasting effect from transfected cells. Cytokines are chosen as genetic adjuvants because they regulate cells involved in host defense and can be used to modulate immune responses. Co-delivery of cytokines in DNA vaccine formulation has been used extensively for a wide range of infectious and parasitic diseases (see Table 2) to enhance the T cell subset responses known to be protective. Vaccine development against schistosomiasis has been hindered by a lack of consensus on the type of immune response that would be protective. However, it is generally believed that the best strategy for an anti-pathology vaccine is immune deviation. Pathology in schistosomiasis is associated with egg-induced granuloma formation for which there is evidence for a role for Th2 cytokines. The strategy here is to use genetic adjuvants of the Th1 cytokine subset, like interleukin-12 (IL-12), to skew the immune response and provide protection [40]. Therefore immune deviation is attained with the use of selected genetic adjuvants.

Siddiqui *et al*. [41] generated DNA vaccines encoding *Schistosoma mansoni *large subunit of calpain (Sm-p80) and either mouse GM-CSF or IL-4 to determine their adjuvant effect in mice. GM-CSF may work as adjuvant through its activating effect on dendritic cells and macrophages. Intramuscular vaccination with Sm-p80 alone provided 39% protection and this protection was significantly increased to 44% with GM-CSF co-administration and 42% with IL-4. The addition of GM-CSF led to an increase in total IgG and IgG1 while Th1 type IgG2a antibody titers remained high in protected animals [42]. Since protection was associated with Th1 type antibodies, the Sm-p80 DNA vaccine was further enhanced with co-delivery of plasmids encoding mouse IL-2 or IL-12 [43]. Greater protection was observed with IL-2 and modest but significantly higher protection was provided by IL-12 co-delivery. Both IL-2 and IL-12 are key cytokines in Th1 cell differentiation. The co-delivery of these cytokines increased IgG2a antibody levels and decreased IgG1 levels, indicating that these genetic adjuvants were successful as Th1 enhancers. Other studies reported no enhancement of protection or immune responses when IL-12 was co-injected, but these differences may be attributed to the nature of the vaccine antigen [44].

## Multivalent Vaccines

Another advantage of DNA vaccines is the possibility to integrate several antigens into the plasmid or to administer a mixture of plasmid vectors. The development of multivalent vaccines consisting of several antigens is a novel approach to create broad range protection against different parasite strains and parasite life cycle stages (see Table 2). Parasites are complex organisms with multiple life cycle stages and antigenic variation mechanisms to evade immune system recognition. Furthermore, not all individuals respond to the same antigens in natural infections. Multivalent vaccines have a greater amount of protective epitopes and could be effective in a greater proportion of the population. However, in multivalent vaccines, the optimal association or combination of antigens must be assessed to obtain synergistic effects.

Vaccination studies against leishmaniasis in mice have identified various parasite antigens with varying degrees of protection as protein vaccines. When combined into multivalent DNA vaccines these antigens have the ability to confer complete or enhanced protection. In fact, a DNA vaccine including a mixture of plasmids encoding three antigens, *Leishmania major*-activated C kinase (LACK), thio-specific antioxidant (TSA), and *L. major *stress-inducible protein (LmST11) was able to induce complete and long lasting protection after parasite challenge in mice compared to killed *Leishmania *parasites and rIL-12 [45]. This protection was characterized by reduced parasite load and the recruitment of CD8+ and CD4+ T cells to the site of infection. The same group tested the combination of these antigens and the route of administration to optimize the results of the previous study [46]. It was determined that a cocktail vaccine composed of all three antigens was more effective than LACK alone or LmSt11 and TSA combined. Furthermore, intradermal injection of the plasmid mixture was more effective than intramuscular or subcutaneous injections, reducing the dose of vaccine required five-fold. Another study also demonstrated that prime-boost co-injection of plasmids encoding two different *L. major *cysteine proteinase genes (Cpa/Cpb) was protective and characterized by IFN-γ production by spleen cells, while separate injections were not protective [47]. The cysteine proteinases are expressed at different levels during parasite development and are thought to be involved in modulation of the host response for parasite survival. In this study the cysteine proteinases, only when combined, had the capacity to induce long lasting immunity of the Th1 type. Comparative evaluations of potential protective antigens is necessary to determine optimal DNA vaccine design [48] as the nature of antigens can have important effects on vaccine efficacy.

## Codon Optimization

Interspecies differences in codon usage are a major obstacle in DNA vaccine development. This is due to the fact that DNA vaccines use host cells for transcription and translation of proteins. Every species has a codon bias for which most genes are encoded and this use of selected codons is related to gene expression efficiency. Closely related species use similar codons. However, in cases where there is a great difference in codon usage between the pathogen and mammals, codon optimization may be required. This strategy involves the modification of codon usage for the genes encoded in a DNA vaccine to a suitable codon bias for increased expression in mammals. This method has proved effective in many systems [19,49,50], increasing protein expression *in vitro *and antigen specific responses in vaccinated animals. In our laboratory, we have developed a codon-optimized DNA vaccine encoding a portion of the *Entamoeba histolytica *Gal-lectin [51]. *E. histolytica *genes are rich in A:T codons, whereas mammalian codons are more G:C rich. Protein expression of the *E. histolytica *Gal-lectin protein using the wild type sequence was difficult and stable clones were difficult to obtain in mammalian cells. Codon optimization was performed to ultimately increase protein expression in gerbils, a model for experimental amoebiasis; therefore gerbil codon usage was used to re-write the Gal-lectin *Hg1l *gene. Transfection of Cos-7 cells with the optimized vaccine construct produced a protein which was immunoreactive with a Gal-lectin specific monoclonal antibody (3F4), demonstrating successful expression of this amoebic protein. Upon vaccination with this codon optimized DNA plasmid, mice developed antigen specific antibodies of the Th1 isotype and Gal-lectin specific cellular immune responses.

## Conclusions

In this review, strategies for increased DNA vaccine efficacy against parasitic diseases to date, i.e. prime-boost immunizations, genetic adjuvants, multivalent vaccines and codon optimization, have been discussed. DNA vaccine technology provides the versatility required to separate protective components of immunity from counter-protective responses. As seen with genetic adjuvants, DNA vaccines can focus on the protective cytokines involved and include antigens that stimulate the production of specific cytokines. This allows designing vaccination strategies that are tailored to a particular infection or even a specific stage of infection. Parasitic diseases are complex, involving changes in immunological responses during the course of infection and changes in immunity to stage specific antigens. The advent of optimization strategies with DNA vaccines presents researchers with the tools to design effective vaccines with specific purposes. It is possible to enhance DNA vaccine efficacy, thus increasing immune responses and protection, through the use of these methodologies. However, it is important to note that these strategies need to be adjusted to the parasite system in order to provide the greatest benefit upon vaccination. For example, Sedegah *et al*. [52] reported reduced immunogenicity of multistage *P. falciparum *DNA vaccines when administered as a mixture of plasmids compared to single plasmid injections. Another study, however, demonstrated that a mixture of three plasmids encoding *P. falciparum *blood-stage antigens had no reduction in immunogenecity when co-injected [53]. Therefore many aspects of a DNA vaccine can contribute to its efficacy, and each must be evaluate to understand the interactions between vaccine components. In fact, it is clear that other factors are important in vaccine design, such as the nature of the antigen, the presence of immunostimulatory CpG motifs in the plasmid backbone, the vaccine delivery system or the site of injection [8,9,16,24,32,54].

The method of vaccine delivery is an important variable in vaccination design. DNA vaccination has been successful through a variety of injection routes, including intradermal, intramuscular, and intranasal. Although intramuscular injections are most common and give consistent responses, alternative routes of delivery may be desired depending on the disease model. Mucosal DNA vaccine immunizations, against intestinal parasites for example, are effective to generate mucosal immune responses at the site of infection. For leishmaniasis, where the disease manifests itself as cutaneous lesions, an intradermal injection targeting Langerhans' cells may be optimal [46]. The gene-gun is a unique method of DNA vaccine delivery, which has been used successfully against a variety of parasites. The gene-gun accelerates plasmid-coated gold particles to supersonic speed with helium gas and delivers them to the outer layers of the skin. In reality this vaccine delivery system is being tested alongside intramuscular injections in The Gambia field trials for malaria vaccines in humans. The Powderject^® ^XR1 is a needle-free powder injection system that delivers fine gold particles coated with the vaccine vectors directly into epidermal cells, specifically dendritic cells. This vaccination method is advantageous since it eliminates the cold chain requirement and reduces the chances of needle-borne contamination. Moreover, the gene-gun method is safe and seems as immunogenic as intramuscular injections in these trials [36].

The greatest challenge in designing DNA vaccines against parasites is making the vaccine suitable for humans while providing strong, long lasting immune responses. Many studies in laboratory animals are successful but the results cannot be replicated in humans. The prime-boost strategy has shown the most success as a delivery technique in larger animals or humans. Field trials with prime-boost malaria vaccines are ongoing and will provide experts with insight with regards to the safety and the immune responses required for protection in humans. Meanwhile, other groups are reporting improved responses in mice or larger mammals with other vaccines, suggesting that this vaccination strategy may be applicable to many other parasitic diseases [3,29]. A variety of combinations of other enhancement strategies with prime-boost immunization have been explored, including the use of genetic adjuvants or multivalent plasmids [11,14,15]. Prime-boost immunizations against *Leishmania *parasites in mice have improved cellular immune responses when plasmids expressing IL-12 and IL-18 are co-injected [11]. A DNA prime-protein boost vaccine in monkeys encoding two *P. cynomolgi *antigens (CSP/TRAP) resulted in lower peak parasitemia and higher antibody and cellular responses than controls [13]. Taken together, the techniques described above will allow parasitologists to develop effective DNA vaccines that are designed to target a specific immune response during parasitic infection. The optimized approach provided by DNA vaccine technology will produce vaccines ready for clinical and practical applications, as well as providing a greater understanding of the underlying complexity of immunity in parasitic infections.

## Competing interests

The author(s) declare that they have no competing interests.

## Author's contribution

CI and KC produced the manuscript together. All authors read and approved the final manuscript.
